# Editorial: Implementation of evidence-based treatments for child anxiety and related disorders across diverse contexts

**DOI:** 10.3389/fpsyt.2023.1248996

**Published:** 2023-07-12

**Authors:** Emily M. Becker-Haimes, Juventino Hernandez Rodriguez, Courtney Benjamin Wolk

**Affiliations:** ^1^Department of Psychiatry, Perelman School of Medicine, University of Pennsylvania, Philadelphia, PA, United States; ^2^Hall Mercer Community Mental Health, University of Pennsylvania Health System, Philadelphia, PA, United States; ^3^Department of Psychological Science, University of Texas Rio Grande Valley, Edinburg, TX, United States

**Keywords:** youth mental health services, implementation, evidence-based intervention, exposure therapy, anxiety

Youth anxiety and related disorders, such as obsessive-compulsive disorder and post-traumatic stress disorder, are prevalent (1), and on the rise (2). Ensuring that anxious youth receive timely and evidence-based treatment is critical; left untreated, these disorders are predictive of a host of future negative outcomes including continued or worsening anxiety, depression, substance abuse, and suicide (3–8). Unfortunately, it can take 15 years or more after a problem is recognized for someone with an anxiety or related disorder to connect with evidence-based psychosocial care, and most youth with anxiety who seek treatment will not receive effective care. This is in large part because as many as 90% of practicing mental health clinicians do not routinely deliver exposure-based cognitive behavioral therapy (Ex-CBT) to their clients struggling with anxiety (9–12), which is the gold-standard, first-line, evidence-based practice (EBP) for anxiety and related disorders (13). In other words, Ex-CBT demonstrates a major research to practice gap: while Ex-CBT is one of the treatments for which we have perhaps some of the strongest evidence for its efficacy and effectiveness, it remains one of the least used treatments within routine clinical care. Note we use the term Ex-CBT to refer to any CBT-based protocol that recognizes maladaptive anxious avoidance as a hallmark psychopathological characteristic of an anxiety or related disorder and works to minimize avoidance and safety-seeking behaviors through approach-oriented strategies (e.g., *in vivo* exposures, interoceptive exposure, exposure with response prevention, and prolonged exposure/trauma narratives).

Using insights from implementation science, or the scientific study of how to increase the use of EBPs in routine clinical settings to improve care quality (14), the past few decades have seen advances in efforts to understand the major barriers leading to the underutilization of Ex-CBT. Identified barriers range from concerns about the complex nature of the intervention itself (15) and poor marketing of Ex-CBT to practicing clinicians and families (16), negative beliefs and misconceptions about Ex-CBT held by clinicians (17–20), organizational constraints and intervention delivery challenges (21, 22), and systemic barriers related to factors such as reimbursement rates and limited funding for specialized training (23). These implementation barriers also occur alongside historical underrepresentation of marginalized and minoritized individuals in clinical treatment trials and limited attention to ways of culturally tailoring treatments to increase engagement and effectiveness. Despite increased understanding of *why* Ex-CBT remains so underutilized, efforts to increase Ex-CBT delivery have had only limited or mixed success (10, 24, 25) or remain in early stages of pilot testing (26, 27). This Research Topic is intended to further advance understanding of how to improve implementation of Ex-CBT for pediatric anxiety and related disorders across the diverse contexts in which youth may receive care, such as outpatient mental health, primary care, and schools.

The varied topics published in this special Research Topic highlight the many ways that researchers are attempting to address the challenge of how to ensure youth with anxiety and related disorders receive the highest quality treatment service. Several articles in this series focus specifically on the need to adapt existing models of Ex-CBT to better fit non-specialty contexts as well as better align the content and format of treatment to address the needs of youth who historically have not been well-represented in clinical trials (e.g., those of historically minoritized identities, those with complex comorbidities). For example, Kendall et al. describe how the various ways a single Ex-CBT protocol—the Coping Cat program (28)—can be adapted in a myriad of ways to improve implementation fit across clinical settings. In contrast, Herres et al. posit that Ex-CBT protocols likely need to be integrated with other treatments drawn from family systems protocols to truly address the complex symptom presentation that many youth present with in community settings. Building on work by others suggesting we should be co-developing novel protocols in tandem with local context leaders to enhance implementabilty and scalability of treatments (29), rather than relying on extant protocols, Gellatly et al. describe the complexity of this process, underscoring the need to collaborate with local context leaders and the critical importance of cultural and contextual considerations to support successful protocol design. Underscoring the importance of treatment adaptation research is work led by Lawson et al. empirically demonstrating the cost-effectiveness of a culturally adapted version of school-based Ex-CBT relative to an unadapted Ex-CBT model.

In an alternative approach, Frank et al. highlight the importance of speaking with caregivers of anxious youth to understand the family experience of trying to access Ex-CBT. Their work suggested several promising implementation strategies targeted directly to consumers that could expedite families' access to quality services. Remaining studies in this Research Topic focused directly on how to best support clinicians to deliver Ex-CBT with fidelity to optimize outcomes. Meza et al. highlight specific supervisory strategies that are associated with improved clinician delivery of exposure-based techniques for youth experiencing symptoms of post-traumatic stress, while Kemp et al. discuss the potential of a novel experiential training strategy (“exposure to exposure”) to directly address the negative beliefs many clinicians hold about Ex-CBT.

Taken together, this Research Topic highlights the importance of adapting Ex-CBT protocols to improve their cultural responsiveness and implementation fit, the critical importance of including patient and family voices in designing implementation strategies to improve Ex-CBT uptake, and the continued need for testing novel strategies that directly address known barriers to Ex-CBT implementation. At the same time, this Research Topic highlights the extraordinary amount of remaining work to be done to truly increase the accessibility and effectiveness of Ex-CBT to all youth who could benefit. In particular, the field will benefit from increasing clarity on how to optimize the cultural responsiveness of Ex-CBT and how and when to sequence Ex-CBT with other treatment models to optimize innovation fit to the increasingly varied settings in which youth seek treatment. This Research Topic also highlighted several promising implementation strategies (e.g., targeted supervisory support, exposure to exposure) ripe for testing in confirmatory hybrid effectiveness-implementation trials. Given the current children's mental health crisis (30), we urge a continued focus on research in this area to alleviate the distress and burden experienced by anxious youth and their families.

## Author contributions

EB-H drafted the initial version. JH and CW provided input and revision to manuscript content. All authors contributed to the conceptualization of content for this manuscript.
